# *trips4health*: Protocol of a single-blinded randomised controlled trial incentivising adults to use public transport for physical activity gain

**DOI:** 10.1016/j.conctc.2020.100619

**Published:** 2020-07-15

**Authors:** M.J. Sharman, K. Ball, S. Greaves, K.A. Jose, M. Morse, C.L. Blizzard, G. Wells, A.J. Venn, A.J. Palmer, D. Lester, J. Williams, S. Harpur, V.J. Cleland

**Affiliations:** a Menzies Institute for Medical Research, University of Tasmania, Hobart, Tasmania, Australia; b Institute for Physical Activity and Nutrition, Deakin University, Geelong, Victoria, Australia; c Institute of Transport and Logistic Studies, The University of Sydney, Sydney, New South Wales, Australia; d Metro Tasmania, Hobart, Tasmania, Australia; e Local Government Association of Tasmania, Hobart, Tasmania, Australia; f Department of Health, Tasmanian Government, Hobart, Tasmania, Australia

**Keywords:** Environment and public health, Health, Public health, Public policy, Exercise, Transportation facilities

## Abstract

**Background:**

Public transport (PT) users typically accumulate more physical activity (PA) than private motor vehicle users yet redressing physical inactivity through transport-related PA (TRPA) interventions has received limited attention. Further, incentive-based strategies can increase leisure-time PA but their impact on TRPA, is unclear. This study's objective is to determine the impact of an incentive-based strategy on TRPA in a regional Australian setting.

**Methods:**

*trips4health* is a single-blinded randomised controlled trial with a four-month intervention phase and subsequent six-month maintenance phase. Participants will be randomised to: an incentives-based intervention (bus trip credit for reaching bus trip targets, weekly text messages to support greater bus use, written PA guidelines); or an active control (written PA guidelines only). Three hundred and fifty adults (≥18 years) from southern Tasmania will be recruited through convenience methods, provide informed consent and baseline information, then be randomised. The primary outcome is change in accelerometer measured average daily step count at baseline and four- and ten-months later. Secondary outcomes are changes in: measured and self-reported travel behaviour (e.g. PT use), PA, sedentary behaviour; self-reported and measured (blood pressure, waist circumference, height, weight) health; travel behaviour perspectives (e.g. enablers/barriers); quality of life; and transport-related costs. Linear mixed model regression will determine group differences. Participant and PT provider level process evaluations will be conducted and intervention costs to the provider determined.

**Discussion:**

*trips4health* will determine the effectiveness of an incentive-based strategy to increase TRPA by targeting PT use. The findings will enable evidence-informed decisions about the worthwhileness of such strategies.

**Trial registration:**

ACTRN12619001136190.

**Universal trial number:**

U1111-1233-8050.

## 1 Background

Physical inactivity is one of the most significant global health concerns, causing 6–10% of coronary heart disease, type 2 diabetes and breast and colon cancers [1]. Physical inactivity poses a similar risk for premature mortality as smoking and obesity and in 2013 was estimated to cost the international community in productivity losses and health care system costs INT$67.5 billion [1,2]. Despite abundant initiatives to support population level increases in physical activity (PA) (e.g. “Life. Be in it”, “This Girl Can”, “International Walk to School Day”), little progress has been made. For instance, the proportion of Australians (aged 15 years and older) adhering to PA guidelines is basically unchanged since the 1980s at around 35–40% [3]. Initiatives to redress physical inactivity have principally focused on leisure-time and sporting activities, with much less attention given to other domains of PA such as transport [4].

Many international, national and state-based frameworks for PA promotion highlight the important impact that active travel (e.g. cycling, walking) supportive environments can have on daily PA [[5], [6], [7], [8]]. Active commuters have significantly lower cardiovascular risk than passive commuters [9] and up to 33 min/day of PA is accumulated through use of public transport according to a recent systematic review [10]. Transport-related PA represents an attractive but under-explored opportunity to support individuals to make small changes that collectively can positively impact population health. While transport-related PA is discretionary, travelling from place to place is commonly a necessary daily behaviour. Drawing on dual process theory, individuals may be more likely to persist with regular PA if it is associated with a habitual pattern, such as travel [[11], [12], [13]].

Incentive-based strategies have demonstrated some promise for improving health behaviours in adults, but relatively few high-quality studies have focused on PA and none specifically on incentivising public transport use for PA gain [[14], [15], [16], [17], [18], [19], [20], [21]]. In a 2017 survey on travel behaviour and health involving 1091 Australian adults, 42.4% of participants rated an incentive-based strategy as likely/extremely likely to increase their public transport use, ranking it as one of the top three public transport use enhancement strategies in the survey [22]. How best to implement incentive-based strategies to increase public transport use for PA gain in real-world settings is unestablished. Behavioural economics theory purports that humans suffer from ‘present bias’, and therefore immediate rather than delayed gratification may be more likely to change behaviour (23,24). Behaviour change may also be more likely when incentives are frequent and progressively escalated [20,23,24]. Incentive-based strategies are also more likely to succeed when supported by other proven behaviour change techniques (e.g. goal-setting, self-monitoring, social support [25]).

*trips4health* is a single-blinded randomised controlled trial designed to fill a knowledge gap and respond to a stakeholder identified need regarding the impact of an incentive-based strategy on public transport use for PA gain. The study findings will benefit service providers, policymakers and practitioners.

## 2 Objectives

The aim of *trips4health* is to establish the impact of an incentive-based strategy (rewarding greater public transport use) on transport-related PA.

## 3 Trial design

Single-blinded controlled trial.

## 4 Materials and methods

Participants, interventions and outcomes.

## 5 Study setting

**Fig. 1:**
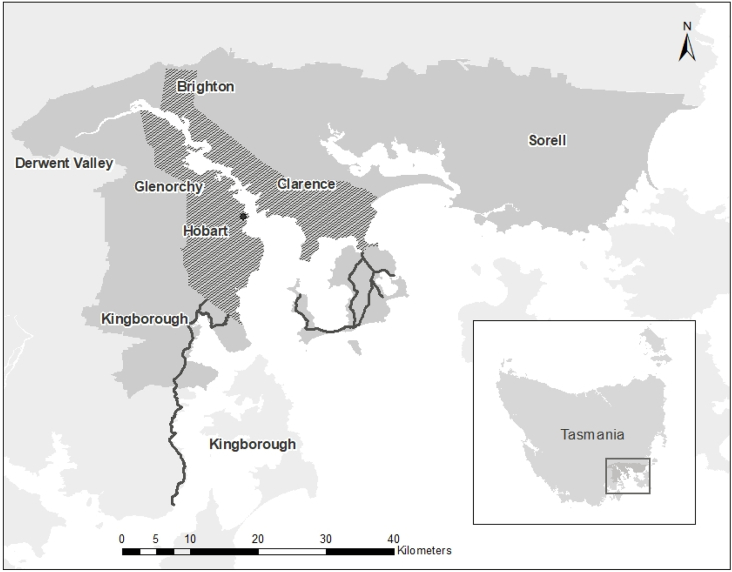
Study Setting: The Greater Hobart area with public transport accessibility identified. Hobart city;  Urban public transport zone;  Non-urban public transport zones;  Greater Hobart Region.

The study will be implemented within the greater area of Hobart, the state capital of Tasmania (Fig. 1). Tasmania is a regional island state of Australia with approximately 510,000 people of which just under half live in the study area [26]. Tasmania has some of the highest rates of chronic disease in Australia and is characterised by high levels of socioeconomic disadvantage [27]. The only mode of public transport is bus, with metropolitan services predominantly offered by one provider (Fig. 1). In 2016, 5.3% of employed residents within the greater Hobart area were estimated to commute by bus as a single method of transport, compared with 76% estimated to commute by driving a car [28].

## 6 Eligibility criteria

**Fig. 2:**
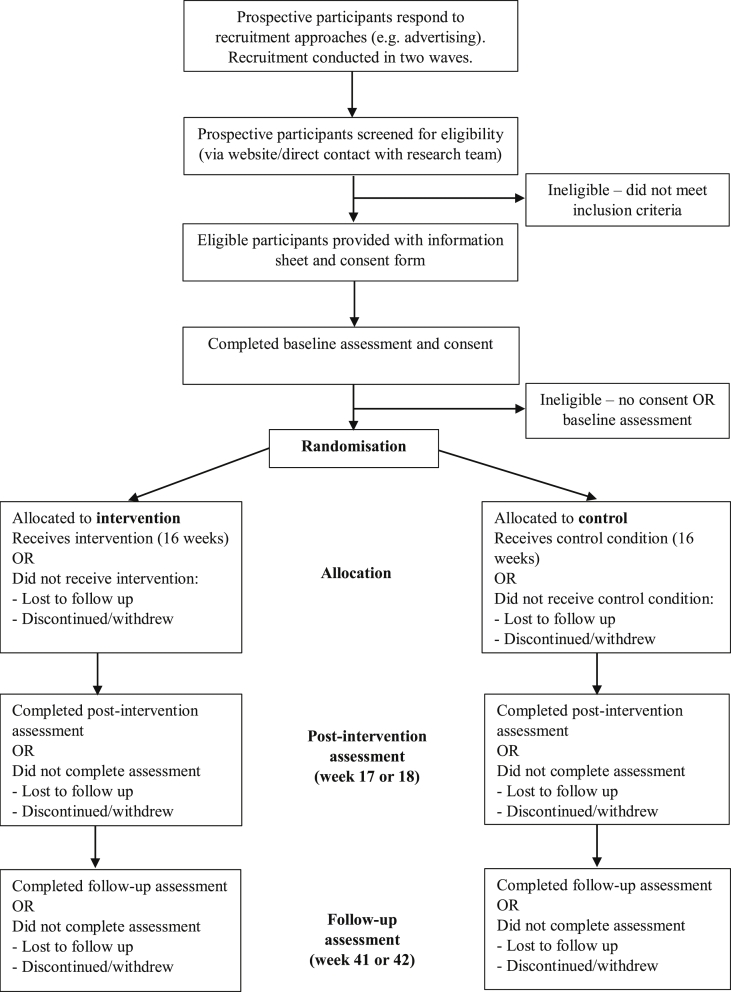
Overview of participant timeline.

Prospective participants’ eligibility will be determined via an online or telephone screening questionnaire (Fig. 2). Inclusion criteria include: ≥ 18 years old; sufficient English proficiency to provide informed consent; living in Southern Tasmania; able to access an urban Tasmania bus service (participant defined); making trips by motor vehicle that could be made by bus; current infrequent bus user (on average ≤ 2 trips per week in the past six months); possession or willingness to possess a public transport smartcard; willingness for the public transport provider and the researchers to access smartcard data; possession of a mobile phone. Exclusion criteria include: intending to move house or work location whereby an urban bus service in Southern Tasmania will be inaccessible within the 10-month study period; currently engaged in or planning to engage in other incentive-based programs to enhance public transport use; pregnancy; a health condition that prevents walking; a health condition that prevents bus use; and a planned activity that would prevent bus use for greater than two weeks during the four month intervention phase of the 10 month study period e.g. surgery, extended holiday.

## 7 Interventions

The *trips4health* study has a four-month intervention phase followed by a six month maintenance phase (Fig. 2). While there is debate in the literature about the minimum length of time needed for habits to form [29] a four-month intervention phase was selected as it allows three months for behaviour change to reach automaticity and one month for maintenance or ‘tapering off’, where contact and support is gradually withdrawn in preparation for intervention cessation [30] (Table 1). Using a ‘gain-framed’ approach (i.e. rewarding positive behaviours), participants in the intervention group will have bus trip targets from weeks 1–16 which escalate over the course of the intervention so that targets become more difficult to reach but incentive values increase. If the bus trip target is met (confirmed through objective smartcard data), participants receive bus trip credit (through their smartcard). The credit received will be commensurate with the participant's default/usual fare type that they were required to set when applying for their smartcard. The price range (AUD$) of the different fare types is $1.92 to $5.76 (a concessional fare through to a full-fare with travel across multiple zones). The maximum credit a participant can receive by the end of the 16-week intervention phase is $103.68 if travelling on a concessional fare and if travelling on a full-fare either $151.20, $207.36 or $311.04 as per their default fare type. Participants will be notified by a weekly email whether they have met or have not met their target, the smartcard credit they have received (if any) and the following week's target. Participants will be aiming to achieve five one-way bus trips per week by the end of the intervention. To assist participants to achieve the weekly targets and, consistent with best practice, incentives will be supported by other behaviour change techniques (e.g. information on consequences of behaviour to the individual, setting graded tasks, goal setting, social support), delivered via weekly mobile text messages [31]. Development of the text messages was underpinned by the Behaviour Change Technique Taxonomy, informed by our previous qualitative work on transport behaviour and PA and from an incentive-based study designed to increase weekly leisure-time PA and reduce sitting time [14,22,32]. Text messages will be offered to the intervention group only. This can help to counter the potential risk that incentives alone may undermine intrinsic motivation in the absence of broader behavioural support. It has been argued that incentives aimed at addressing habitual behaviours such as physical inactivity may be more impactful if delivered as part of effective behaviour change programs [31]. Both the intervention and control groups will receive printed versions of Australia's Physical Activity and Sedentary Behaviour Guidelines [33]. Educational materials have been selected as an active control to help engage and retain the control group – alone, this type of cognitive strategy is unlikely to influence PA behaviour [34]. All intervention support will be provided by a member of the research team.

**Table 1:** *trips4health* intervention schedule.

	Intervention phase	Bus trip targets	Smartcard credit available (all zones full-fare^a^)	Smartcard credit available (all zones concessional fare^b^)	Text message frequency
Within 2 weeks pre-intervention	*Baseline survey, smartcard data, clinic assessment, accelerometer, travel behaviour smartphone app*				
Week 1	Getting started	1	$5.76	$1.92	x 2
Week 2	Getting started	1	$5.76	$1.92	x 2
Week 3	Slowly increasing	2	$11.52	$3.84	x 2
Week 4	Slowly increasing	2	$11.52	$3.84	x 2
Week 5	Moving along	3	$17.28	$5.76	x 2
Week 6	Moving along	3	$17.28	$5.76	x 2
Week 7	Moving along	3	$17.28	$5.76	x 2
Week 8	Moving along	3	$17.28	$5.76	x 2
Week 9	Aiming high	4	$23.04	$7.68	x 2
Week 10	Aiming high	4	$23.04	$7.68	x 2
Week 11	Aiming high	4	$23.04	$7.68	x 2
Week 12	Aiming high	4	$23.04	$7.68	x 2
Week 13	Keeping up	5	$28.80	$9.60	x 1
Week 14	Keeping up	5	$28.80	$9.60	x 1
Week 15	Keeping up	5	$28.80	$9.60	x 1
Week 16	Keeping up	5	$28.80	$9.60	x 1
***Total***		**54**	**$311.04** (AUD)	**$103.68** (AUD)	**28**
Week 17 or 18	*Post-intervention survey, smartcard data, clinic assessment, accelerometer, travel behaviour smartphone app*				
Week 41 or 42 >(10 months from baseline)	*Follow-up survey, smartcard data, clinic assessment, accelerometer, travel behaviour smartphone app*				

a most expensive fare type.

b cheapest fare type. 50% of the public transport provider's adult passengers travel on a concessional fare.

## 8 Outcomes

The primary outcome is change in average daily step count measured by accelerometer over seven days at baseline (Timepoint 1/T1), immediately post-intervention (week 17/18; Timepoint 2/T2; primary endpoint) and at the end of the maintenance phase (week 41/42; Timepoint 3/T3). The secondary outcomes are change in: self-reported and measured (via a smartphone app collected over seven days and smartcard data) travel behaviour (e.g. mode (e.g. motor vehicle), frequency and duration); perspectives on travel behaviour (e.g. enablers and barriers); self-reported out of pocket transport-related expenses; self-reported work productivity; self-reported commute time to work/study location; self-reported commute time by different transport modes; self-reported PA (transport, leisure, occupational, domestic, total) (mins/week) and sedentary behaviour (sitting) using the International Physical Activity Questionnaire – Long form (IPAQ – L) supplemented with additional questions in the study-specific survey [35]; accelerometer measured minutes/week of PA, sedentary behaviour and PA intensity; measured blood pressure using a validated automated blood pressure device and waist circumference determined by a measurement tape; body mass index calculated from measured (using a stadiometer and digital scales) or self-reported height and weight; and self-reported health, with quality of life (QoL) measured by the Assessment of Quality of Life (AQoL)-8D [36,37]. Costs incurred by the public transport provider to implement the intervention will be measured by data supplied by the provider at trial completion. All self-reported outcomes will be measured through the study-specific survey at all three timepoints.

## 9 Sample size

Sample size calculations are based on the comparison of the primary outcome in the control versus intervention group. The calculations are based on mean and standard deviation of daily steps as measured by accelerometer (8000 ± 3500) based on data from a sample of participants using the same measure [38]. The assumed correlation between baseline and at 10 months follow-up was 0.7, with a high level of stability expected between baseline and follow-up because PA typically tracks well over shorter periods of time (e.g. weeks and months) [39]. A high level of stability is expected between baseline and at 10 months follow-up because the unit of analysis will be based on average daily steps. A total sample of 300 participants would provide 80% power with α = 0.05 to detect a difference of 624 steps/day (an 8% change). Allowing for expected attrition (based on a previous related PA incentives-based study [14]), the plan is to recruit 350 participants.

## 10 Recruitment

Participants will be recruited through social/traditional media, workplaces, word-of-mouth, bus advertising, professional networks, and co-promotion through other related projects. To minimise self-selection based on a preference for PA, promotional materials will be framed to focus on health generally, noting the potential for smartcard credit (i.e. ‘Does taking the bus = better health? Want to earn over 50 free bus trips?’). Recruitment materials will direct potential participants to a study website and provide email and telephone contact details. The study website will provide more detailed information, including a link to the screening survey.

The recruitment phase will occur in two waves, with Wave 1 commencing in September 2019 and Wave 2 commencing in February 2020. The reason for conducting recruitment in two stages is to reduce resource burden and to lessen the impact of seasonal variation in PA and public transport use. Each recruitment wave is expected to take up to three months but will be extended if necessary. The positive response (n = 1091) with minimal advertising in a two-week period in our 2017 transport and physical activity behaviour project indicates high feasibility of this approach [22]. Participants will be directed to register their interest via the study website page, email or telephone contact. To facilitate recruitment, all participants (irrespective of group allocation) will receive smartcard credit to a maximum value of $30 (AUD) as study compensation. Compensation will be commensurate with participation and is independent of the incentives scheme: completion of the baseline assessment (T1) = $5 credit; completion of the post-intervention assessment (T2) = $10 credit; and completion of the follow-up assessment (T3) = $15 credit. Greater compensation for greater participation will be employed as a strategy to enhance retention and mitigate drop-out, particularly among the control group. This small amount of compensation was considered unlikely to alter usual travel behaviour.

## 11 Assignment of interventions

### 11.1 Allocation

After completion of the baseline assessment (T1), participants will be randomly allocated to the control or intervention arm. The allocation sequence will be created using computer-generated random numbers on a 1:1 ratio without stratification. Randomisation will be conducted in blocks of four, the details of which will be unavailable to research team members who enrol participants in the respective study arms.

### 11.2 Blinding

Due to the nature of the intervention, participants cannot be blinded to treatment allocation. All members of the research team will be blinded except for two research assistants (RAs). The unblinded RAs will be responsible for enrolling participants, assigning participants to the control or intervention arms according to the treatment allocation sequence generated through the software Research Electronic Data Capture (REDCap, Version 8.5.19, Nashville, Tennessee, USA), informing participants of the requirements of being in the respective study arm, advising participants in the intervention arm if they have met their weekly bus trip target and the subsequent weekly target, coordinating clinic assessments and overall be the main contact point for participants. The unblinded RAs will not be involved in conducting the post randomisation clinic assessments at T2 and T3 or data analysis. The research team member who creates the randomisation sequence will have no contact with participants and will not be involved with data collection or analysis. There are no foreseen circumstances by which unblinding of other research team members would need to occur.

## 12 Data collection, management and statistical methods

### 12.1 Data collection methods

All participants will complete a baseline assessment (T1), an assessment immediately post-intervention (4 months post intervention commencement, T2) and a follow-up assessment six months post-intervention (10 months post-intervention commencement, T3) to assess if any changes are sustained once incentives are ceased. To maximise participation and ensure acceptable levels of participant burden, a stepped approach will ensure a minimum amount of data collection from all participants, with more burdensome measures related to the secondary outcomes from those willing and able to do so (Table 2). Surveys will be offered electronically, with hard copies provided only if necessary. The survey will include demographic, travel, PA, health and economic related questions. The IPAQ-L is included in the survey because it is reliable, widely used and separates leisure and transport-related PA [35]. The AQoL–8D is a reliable and valid QoL evaluation tool, has Australian population norms, is sensitive to minor changes in QoL and has been included in the survey to complement the health-related questions and to attain health state utility values [36,37]. Permission to use participants' smartcard data is an eligibility requirement and will be sought during the screening and consent process. Data collected from the smartcard includes the date and time (at point of boarding only) of each trip. Smartcard data will be retrieved for the two months prior to commencement of the study (for participants with an existing smartcard) to enable objective assessment of participants’ usual bus use. Participants with a smartphone will be asked to download the state-of-the-art *trips4health* smartphone app. The app uses Global Positioning System (GPS) technology to passively track travel behaviour and automatically infer trips, together with start/end times and travel duration. The app is designed to overcome known limitations of traditional self-report methods (e.g., travel diaries), which are highly burdensome, and among other limitations, poor at picking up short, incidental active transport trips, a critical issue for this study [40]. Participants are prompted each day over a seven-day period to confirm trips are genuine and provide travel mode and purpose through a simple interface with each trip taking a few seconds to code. The app will be introduced at the first clinic assessment, providing a unique face-to-face opportunity to explain how the app works as well as easing known barriers, particularly around privacy.

**Table 2:** Stepped levels of assessment participation.

Level of assessment	Survey	Smartcard data	Accelerometer	*trips4health* app	Physical measures
Minimum	✓	✓	✓		
Medium	✓	✓	✓	✓	
	✓	✓	✓		✓
High	✓	✓	✓	✓	✓

Physical measurements (blood pressure, height, weight, waist circumference) will be taken and accelerometers (ActiGraph GT3X and GT3X+) administered at the clinic assessments. All clinic assessments will be conducted at a single clinical research facility. To ensure high quality data collection, respective research team members will be trained according to standardised protocols. Height will be measured using a fixed stadiometer to the nearest 0.1 cm, and weight measured to the nearest 0.1 kg. Waist circumference will be measured at the level of the narrowest point between the lower costal (10th rib) border and the iliac crest using a Figure Finder tape measure. Three blood pressure readings at 1-min intervals will be taken after 5 min of rest (feet flat on the floor, legs uncrossed, back supported, shoulders and arms relaxed, forearms resting on chair/table) using an automated Omron HEM 907 blood pressure device.

Participants will be asked to wear a wrist-worn accelerometer for seven consecutive days, as recommended [41]. Accelerometers accurately and reliably measure step count, PA frequency, intensity and duration and sedentary behaviour [[42], [43], [44]]. Accelerometers will be issued where possible at clinic assessments, but for those unable or unwilling to attend a clinic, accelerometers will be mailed to participants in padded postage packs with instructions provided via telephone. As well as verbal instructions, all participants will receive detailed written instructions for wearing the accelerometer including monitor placement, how to return the monitor via a reply-paid padded postage pack and contact details of the study team, and a brief diary for noting the date of wear and any notable comments (e.g. unwell, forgot to wear). Monitors not returned will be followed up via telephone and email until received.

### 12.2 Data management

Participants will complete the survey through REDCap. Physical measurements will be entered into REDCap by a trained research team member. To ensure data quality, range and aberrant data value checks will be set up in REDCap to highlight possible errors. Electronic data collected through the app will be stored in an isolated, single purpose, secure database hosted by Tourism Research Technology (Tasmania, Australia), behind a firewall with limited and restricted organisational access and with appropriate deployment, security and permissions configurations. Once downloaded by the research team, the app data along with all other data will be stored on a secure, password-protected database behind University firewalls. Only members of the research team involved in data analysis will have access to the research data. This study is part of an intended program of work that will take place over several years. For this reason, the data will be kept indefinitely and for no less than 5 years. Participants can give optional consent for indefinite storage of their data. Any data destruction will be via deletion of electronic files. Deidentified data may be used by researchers outside of the investigator team provided that due ethics processes are adhered to.

### 12.3 Statistical methods

Quantitative Evaluation: Descriptive statistics (means and proportions) will characterise the outcomes according to group allocation. Participant characteristics will be described and compared across groups. In the unlikely event that there is an uneven distribution of participant characteristics (e.g. gender, age, education) across the control and intervention groups resulting from chance (rather than bias) [45], consideration will be given to clinical meaningfulness and the size of any imbalances that may have occurred [46]. Comparisons will also be made with the general population with the purpose of informing conclusions about the generalisability of the participant groups. Linear mixed model (LMM) regression will compare differences in average daily step count (primary outcome) between control and intervention groups. LMM will also be used to compare differences between the control and intervention groups regarding the secondary outcomes. LMMs allow for missing outcome data and provide methods to account for correlated observations. Analyses will be undertaken on an intention-to-treat basis.

Accelerometer data will be collected using 60-s epochs, and Freedson 1998 equations used to determine cutpoints for sedentary, light, moderate and vigorous intensity physical activities [47]. The impact of applying the criteria of minimum daily wear time (10 h/day), minimum number of days worn (4 days) and minimum number of weekdays and weekend days (3 weekdays and 1 weekend day), as well as consideration of daily wear time values, will be examined in sensitivity analyses [48].

Self-reported domain-specific data from the IPAQ will enable examination of any displacement of leisure-time PA for time spent on public transport and/or in transport-related PA, which could potentially explain null findings.

Secondary analyses to better understand possible enabling factors that may influence adherence to protocols and intervention effectiveness will be undertaken so that appropriate recommendations can be made about future rollout or upscaling of the intervention.

Economic Evaluation: Non-research related resources associated with the implementation of the intervention will be documented and the comparative costs and benefits of the intervention relative to the control group will be assessed. The primary outcome of the economic analysis is a cost comparison between intervention and control groups, that is, a cost-benefit analysis with all costs and benefits expressed in monetary (AUD$) terms.

Modelled analyses will be undertaken to assess the financial implications of broader implementation rollout and intervention upscaling. Personal time (e.g. more/less time spent commuting), productivity and outlay (e.g. walking equipment, fuel, parking) costs will also be evaluated.

Process Evaluation: This will assess the fidelity and quality of implementation, clarify causal mechanisms and identify contextual factors associated with outcome variation [49]. Using the Medical Research Council UK's framework [50] designed for examining complex public health interventions, intervention acceptability at completion of the intervention will be assessed (T2) through face-to-face or telephone interviews with intervention group participants (n = 20 or until saturation of major themes) who did and did not reach bus trip targets. Participants in the intervention arm will be asked if they are willing to participate in an interview when completing the survey at T2. Interviews will be timed to coincide with clinic attendance. The recruitment and implementation impact for the public transport provider will be determined through face-to-face interviews or group discussion with key personnel (n = ≤5) at key points during the implementation of the intervention (i.e. intervention development, mid-intervention and intervention completion). Process evaluation items will be included in all three surveys for all intervention group participants.

## 13 Monitoring

### 13.1 Data monitoring

*trips4health* is a behaviour change study that does not require participants to undertake risky clinical procedures or medication regimens. For this reason, a Data Monitoring Committee was considered unnecessary, likewise the development of an interim analysis plan and stopping guidelines.

### 13.2 Harms

PA intervention studies pose minimal risk to participants [51]. Although unlikely, the most common PA intervention study-related adverse events are minor musculoskeletal injuries [51]. Adverse events reported to members of the research team will be logged in REDCap, reviewed by the chief investigator to determine the appropriate action and reported to the relevant ethics committee.

At study completion, participants will be provided with a feedback letter summarising their health information (including PA, body mass index, waist circumference, blood pressure). Participants will be encouraged to share this summary information with their health care practitioner, especially if values are outside normal limits. Participants will only be provided with information about their health at the clinic assessments if their blood pressure exceeds 140/90 mmHg and under such circumstances they will be encouraged to see their health practitioner.

### 13.3 Auditing

After approximately 10% of the total sample have completed week two of the intervention phase, an unblinded member of the research team will conduct an audit to ensure all documentation has been completed and appropriately managed and that the study is being operationalised correctly (e.g. consent forms signed and stored, randomisation process working, surveys complete, data stored appropriately, correspondence accurate and timely, bus trip incentives correctly allocated).

## 14 Ethics and dissemination

### 14.1 Protocol amendments

Any changes made to the protocol will be submitted to the Tasmanian Health and Medical Human Research Ethics Committee for approval. Relevant parties will be alerted to protocol changes where necessary. Participants will be asked to re-consent if any protocol changes are made that may directly impact them (e.g. changed eligibility criteria).

### 14.2 Consent or assent

Participants will provide consent electronically through REDCap or in person with research staff when attending the first clinic (T1). Consent forms will be stored with study data.

### 14.3 Confidentiality

Access to administrative data (e.g. names, telephone numbers, email addresses) will be limited to research team members on a need to know basis. For the purpose of data cleaning and analysis, research data will be kept separately from administrative information. Participants will be assigned a unique identifier and only the unique identifier will be used throughout data cleaning and analysis. Only investigators will have access to the final de-identified trial data. Reports, publications and presentations arising from the study will not include any information that would enable the identification of participants.

### 14.4 Dissemination policy

Participants will have the option of being provided with a summary of findings and alerted to any publicly available reports produced. At the end of the study they will also be offered summary personal information.

Findings will be disseminated through traditional academic pathways including conferences and peer-reviewed journals. A strategic and inclusive knowledge transfer strategy will ensure that findings are disseminated through appropriate professional mechanisms (e.g. State government departments, local government, advisory councils, national PA networks) and delivered in formats to maximise policy and practice impact and community benefit [52]. The knowledge transfer strategy will be developed in consultation with key stakeholders.

## 15 Discussion

*trips4health* responds to calls for more trial evidence examining the relationship between PA and transport behaviour and PA interventions that can be scaled up [4,53]. It will also address lack of investigation into the role of incentives-based interventions for increasing PA, particularly transport-related PA.

The comprehensive analysis framework includes both objective and subjective measures of travel and PA behaviour (including mediators), economics, health economics and process evaluation which will result in a rich data set, enabling evidence-based decision making within policy and practice settings. The use of an implementation science framework is another strength of the study design and will ensure that key stakeholders receive the information they need and in the desired format to implement the findings for maximum effect [52].

Physical inactivity is pandemic, causing substantive individual and social impact. Redressing population level physical inactivity is widely prioritised but efforts to correct the behaviour have predominantly focused on leisure-time PA and have been largely unsuccessful. *trips4health* addresses a research gap by investigating novel and scalable ways to increase PA in real-world settings.

## Ethics approval and consent to participate

Ethics approval was granted by the Tasmanian Health and Medical Human Research Ethics Committee (approval number H0017820, 27 March 2019). Consent will be obtained from all participants.

## Consent for publication

Not applicable.

## Availability of data and material

The datasets used and/or analysed during the current study are available from the corresponding author on reasonable request with appropriate approvals.

## Funding

The study was funded by a 10.13039/501100000925National Health and Medical Research Council Partnership Project (APP1152999). VC is supported by a 10.13039/501100001030National Heart Foundation of Australia Future Leader Fellowship (2016–2020, ID 100444).

## Declaration of competing interest

The authors declare that they have no competing interests.
